# Ventilator-Associated Pneumonia (VAP) Prevention Bundle: A Multicenter Cross-Sectional Saudi Study to Assess Knowledge, Adherence, and Perceived Barriers Among ICU Practitioners in Hail Region

**DOI:** 10.3390/pathogens15060656

**Published:** 2026-06-22

**Authors:** Ashwaq Abdullah Alanezi, Waleed E. Elawamy, Huda Khalaf Alshammri, Eman Ali Elkordy, Ahmed E. Taha

**Affiliations:** 1 Infection Prevention and Control Department, Hail Health Cluster, Ministry of Health (MOH), Hail 81411, Hail Province, Saudi Arabia; aalanazi401@moh.gov.sa; 2 Microbiology and Immunology Unit (Parasitology Division), Department of Pathology, College of Medicine, Jouf University, Sakaka 72388, Aljouf Province, Saudi Arabia; welawamy@ju.edu.sa; 3 Department of Medical Parasitology, Faculty of Medicine, Benha University, Benha 13511, Al-Qalyubiyya Governorate, Egypt; 4 Infection Control Directorate at the Hail Health Cluster, Ministry of Health (MOH), Hail 81411, Hail Province, Saudi Arabia; hukalshammari@moh.gov.sa; 5 Department of Anatomy & Physiology, College of Medicine, Imam Mohammad Ibn Saud Islamic University (IMSIU), Riyadh 11461, Riyadh Province, Saudi Arabia; 6 Microbiology and Immunology Unit, Department of Pathology, College of Medicine, Jouf University, Sakaka 72388, Aljouf Province, Saudi Arabia; 7 Department of Medical Microbiology and Immunology, Faculty of Medicine, Mansoura University, Mansoura 35516, Aldakahlia Governorate, Egypt

**Keywords:** ARDS, CDC, ICU, healthcare-associated infection, healthcare workers, mechanical ventilation, ventilator-associated events, ventilator-associated pneumonia

## Abstract

Ventilator-associated pneumonia (VAP) is linked to high mortality rates, especially in developing countries. This cross-sectional survey study was conducted across three central hospitals in the Hail region of Saudi Arabia, King Salman Specialist Hospital, Hail General Hospital, and King Khalid Hospital, to assess the knowledge and adherence of intensive care unit (ICU) healthcare practitioners to the ventilator bundle (VB) for VAP prevention. It also looked at the practitioners’ perceived barriers to effective VB deployment. The study (*n* = 86) revealed significant disparities in VAP prevention knowledge across educational levels regarding the recommended degree of head-of-bed (HOB) elevation (*p* < 0.001), the use of endotracheal tubes with extra lumens for subglottic drainage (*p* < 0.001), and the protective effects of 0.12% chlorhexidine gluconate antiseptic oral rinse (*p* = 0.019). Professional experience significantly influenced knowledge of non-standard VB components (*p* < 0.001), the recommended frequency of awakening and spontaneous breathing trials (SBTs) (*p* < 0.001), and knowledge of extra-lumen tubes (*p* = 0.038) and kinetic beds vs. standard beds (*p* = 0.005). Significant differences were found between professional categories regarding knowledge of hand hygiene performance (*p* = 0.032), the correct degree of HOB elevation (*p* = 0.007), and patient positioning (semi-recumbent vs. supine) (*p* = 0.023). Years of experience significantly impacted reported compliance with institutional VB (*p* = 0.013), adherence to oral care protocols (*p* = 0.035), and the assessment of sedation depth (*p* = 0.002). While basic measures like HOB elevation practice and DVT prophylaxis showed universal reported compliance (100%), significant performance gaps were identified in more complex tasks, such as interrupting continuous sedative infusions and performing SBTs as recommended (*p* < 0.001), particularly among novice practitioners. The primary implementation barrier preventing full compliance with the VB was identified as educational deficit, which was prioritized as the most important area for quality improvement, highlighting the need for targeted training for newly hired ICU staff.

## 1. Introduction

Ventilator-associated pneumonia (VAP) is a significant complication that affects patients on mechanical ventilation, with 5% to 40% of them developing infections [1]. VAP is linked to high mortality rates, especially in developing countries, and is routinely ranked as one of the most common healthcare-associated infections (HAIs) in both developed and developing countries. In addition to its severe outcomes, VAP leads to considerable healthcare resource utilization due to prolonged hospital stays and associated complications [2]. However, studies indicate that up to 50% of VAP cases can be prevented, and the implementation of preventive care ventilator bundle (VB) has proven effective in significantly reducing the risk of VAP in ventilated patients [3]. A bundle is a collection of distinct components used to create a set of quality indicators for a certain system, operation, or treatment. To dramatically improve performance, all these interventions must be executed together [4].

The Centers for Disease Control and Prevention (CDC) defined a VAE as a sustained rise in ventilator support after a phase in which it was stable or decreasing. This definition is implemented by monitoring two ventilator parameters: positive end-expiratory pressure (PEEP) and the fraction of inspired oxygen (FIO_2_). Operationally, a VAE is identified when either (1) there are at least two baseline days showing stable or decreasing daily minimum PEEP levels, followed by at least two days where the daily minimum PEEP is at least 3 cm H_2_O higher than the corresponding baseline days, or (2) there are at least two baseline days with stable or decreasing daily minimum FIO_2_ levels, followed by at least two days where the daily minimum FIO_2_ is at least 20 points higher than the corresponding baseline days [5].

The VAE criteria also include subcategories intended to distinguish VAEs that may be related to infection, and—within that group—those that could specifically be pneumonia-related. The infection-related subset is termed infection-related ventilator-associated condition (IVAC). An IVAC requires evidence of abnormal body temperature (below 36 °C or above 38 °C) or an abnormal white blood cells count (WBCs ≤ 4000 cells/mm^3^ or ≥12,000 cells/mm^3^), along with at least four days of newly started antibiotics, initiated within two days of the onset of the VAE. “Possible VAP” is then defined either by histopathologic findings or by IVAC cases that also have concurrent positive respiratory cultures or positive microbiological tests for Legionella species or for respiratory viruses [6].

The microbiological requirement can be met in one of two ways. First, a quantitative culture threshold, adjusted to the specimen type, may be exceeded (for example, endotracheal aspirate with ≥10^5^ colony-forming units; CFUs/mL vs. bronchoalveolar lavage with ≥10^4^ CFUs/mL vs. protected specimen brush sample with ≥10^3^ CFUs/mL). Second, the criterion can be satisfied by any detectable amount of growth, provided the Gram stain also shows evidence of purulence, defined as at least 25 neutrophils and no more than 10 epithelial cells per low-power field [7].

The Institute of Healthcare Improvement (IHI) recommended a VB to reduce morbidity and death in VAP patients. Improving patient safety and lowering problems related to mechanical ventilation need the adoption of these strategies including head-of-bed (HOB) elevation to 30 to 45 degrees; daily “sedation vacation” and evaluation of extubation readiness with daily assessment for spontaneous breathing trials; SBTs; prophylaxis for peptic ulcer disease (PUD); prophylaxis for deep vein thrombosis (DVT); and daily oral care with chlorhexidine and subglottic drainage to remove secretions from around the vocal cord and trachea to prevent them from entering the lungs, in addition to appropriate hand hygiene as one of the most important standard precautions for infection prevention [8]. Intensive care units (ICUs) have varied in the VAP bundles’ included interventions, and there is not a single bundle that all communities can agree to use. However, compliance rates with the VAP bundle must be higher than 95% to effectively eradicate VAP cases. Regular long-term commitment to nursing and medical staff performance measurement systems is advised [9].

In fact, certain interventions, such as chlorhexidine-based oral care and stress ulcer prevention, may increase the risk of VAE. New data suggests reducing atelectasis by commencing mechanical ventilation with increased PEEP (8 cm H_2_O), minimizing sedation, performing daily SBTs, implementing conservative fluid management, and avoiding excessive tidal volumes and needless blood transfusions. These measures try to reduce the amount of time spent on mechanical ventilation while also targeting the primary causes of VAEs, such as pneumonia, fluid overload, acute respiratory distress syndrome (ARDS), and atelectasis. Overall, advancing VAE prevention necessitates reviewing existing bundles and implementing evidence-based guidelines (EBGs) focusing on objective metrics and timely care [10].

Using EBGs to provide high-quality treatment is a challenge for critical care nurses. Individual preventive strategies are used in the bundled practices approach to lower the incidence and prevalence of VAP and enhance patient outcomes. The nurses’ inadequate knowledge scores were reported. To lower the prevalence of VAP, training programs for critical care nurses on infection control and VAP bundle preventive measures were recommended [11].

There are still several significant gaps in the growing body of literature addressing VAP preventive and control strategies. First, rather than evaluating healthcare personnel’ adherence to the VB for VAP prevention, much earlier research concentrated on their knowledge and/or attitudes. Healthcare workers’ adherence to EBGs is more accurately reflected in real clinical practices. Assessing knowledge and adherence can help identify barriers to effective VB implementation. Second, healthcare workers’ knowledge, and compliance with VB can vary among healthcare settings and geographical regions. Even within the same geographical region, knowledge and compliance can vary according to educational levels, professional experience, and professional categories of the healthcare providers. Much research focused only on nursing people. VAP prevention is a multidisciplinary task. Thus, this cross-sectional survey study was conducted across three central hospitals in the Hail region of Saudi Arabia, King Salman Specialist Hospital, Hail General Hospital, and King Khalid Hospital, to assess the knowledge and adherence of ICU healthcare practitioners to the VB for VAP prevention. It also looked at the practitioners’ perceived barriers to effective VB deployment.

## 2. Materials and Methods

### 2.1. Ethical Considerations

The Institutional Research Board (IRB) of the Directorate of Health Affairs in Hail in Saudi Arabia approved the study protocol (IRB Log Number: 2025-10) on 10 February 2025. The research adheres to the 1975 Declaration of Helsinki’s ethical standards. There were adequate provisions to maintain the privacy of participants and confidentiality of the data. Participants were informed about the study’s purpose, voluntary participation, data confidentiality, and anonymous processing of replies prior to obtaining the questionnaire. Informed consent was implied through voluntary participation and completion of the questionnaire.

### 2.2. Design of the Study

After securing the IRB approval, a cross-sectional study was carried out to assess knowledge, adherence, and perceived barriers among healthcare practitioners working in adult ICUs regarding the implementation of the VB for preventing VAP during the period between March 2025 and July 2025. The study included the ICUs’ physicians, nurses, and respiratory therapists in the three central hospitals in the Hail region (King Salman Specialist Hospital, Hail General Hospital, and King Khalid Hospital-1) who have responsibility of direct care for adult (≥18 years) ICU patients on mechanical ventilation ≥ 48 h and agreed to participate in the study. The healthcare workers that refused to take part in this research and/or gave incomplete or invalid data were excluded from the study. No missing data imputation was undertaken. The study was specifically developed to assess respondents’ knowledge and adherence based on their level of medical education, years of experience, and job title.

### 2.3. Calculation of Sample Size

The adjusted sample size (finite population) was calculated using an online (Raosoft Incorporation, 2025) sample size calculator (https://raosoftcalculator.com/, accessed on 1 February 2025) with response distribution of 50%, confidence level 95%, margin of error of 5.00%, and 110 target population (ICUs’ physicians, nurses, and respiratory therapists in the three central hospitals in the Hail region; King Salman Specialist Hospital, Hail General Hospital, and King Khalid Hospital-1). The study included 86 participants.

### 2.4. Questionnaire Structure and Collection of Data

The data collection tool in this study was adapted from the validated questionnaire developed by Paliwal et al. [12]. This tool had previously undergone expert review and was specifically designed to evaluate both knowledge and compliance related to the ventilator bundle in the ICU settings. The study team assessed the questionnaire’s content and format, and a statistician revised its statistical design before it was distributed. To maximize participation, the questionnaire was distributed using two methods: it was shared online through secure electronic forms (via a Google Forms link communicated through official hospitals’ channels), and it was also made available for face-to-face completion through interviews conducted by one of the investigators for participants who preferred an in-person format. The participants were recruited through a convenience sampling approach. Using these recruitment methods may lead to selection bias and reduce sample representativeness.

The survey included items on demographics and training experience regarding EBGs for VAP prevention. The questionnaire was divided into two main sections. The first section was designed to assess the level of knowledge of ICU healthcare practitioners regarding EBGs for the prevention of VAP. The questions were structured in a clear and concise format, covering essential aspects of the VB components recommended by the IHI. This allowed for a standardized evaluation of the participants’ knowledge and understanding of recommended preventive practices. The questions included were as follows: Q1: Oral vs. nasal route for endotracheal intubation. Q2: Which of the following is not a standard component of the VB? Q3: Which of the following does not increase the incidence of VAP? Q4: To reduce the risk of VAP, the head end of the bed should be elevated to which degree? Q5: How often should you attempt awakening and spontaneous breathing trials? Q6: How often should you perform hand hygiene? Q7: What is the effect of endotracheal tubes with extra lumen for drainage of subglottic secretions? Q8: Kinetic vs. standard beds. Q9: Patient positioning. Q10: Usage of 0.12% chlorhexidine gluconate antiseptic oral rinse.

The second section of the questionnaire was focused on self-reported adherence to the VB in daily practice as recommended by the IHI. The participants were asked to respond to the following questions, yes or no; Q1: I always comply with the Institutional VB; Q2: I adhere to existing oral care protocol; Q3: I always use chlorhexidine oral rinse as recommended; Q4: I assess the depth of sedation as often as recommended; Q5: I interrupt continuous sedative infusions as recommended; Q6: I perform spontaneous breathing test as recommended; Q7: I always keep head of bed elevated to 30–45°; Q8: I always make sure that mechanical DVT prophylaxis is inserted as recommended; Q9: I always give pharmacological DVT prophylaxis as recommended, and Q10: I always give pharmacological peptic ulcer prophylaxis as recommended. The overall compliance for each VB component was analyzed to identify which components had the highest and lowest compliance, thereby highlighting priority areas for further improvement in ICU VB implementation.

Furthermore, participants were asked to select from a list of predefined barriers when they reported difficulty in implementing specific components. The items in this section were constructed to reflect common challenges encountered in clinical practice, including lack of guidelines, lack of education, inadequate resources, disagreement with reported results, fear of potential adverse effects, patient discomfort and/or high costs.

### 2.5. Statistical Analysis

Data analysis was done by SPSS v28 (IBM Inc., Armonk, NY, USA). Categorical variables were presented as absolute frequencies and percentages. The chi-square test was used to compare the groups for the questionnaire’s categorical variables. A *p* value < 0.05 was considered statistically significant.

## 3. Results

### 3.1. Participants’ Demographic Data

The demographic data of participants were presented in Table 1.

### 3.2. Total Participants’ Responses Expressing Their Knowledge of and Adherence to EBGs for VAP Prevention

A summary of participants’ responses expressing their knowledge of and adherence to the VB for VAP prevention was shown in Table 2.

### 3.3. Effect of Participants’ Educational Level on the Responses Expressing Their Knowledge of and Adherence to EBGs for VAP Prevention

Table 3 summarizes the effect of participants’ educational level on the responses expressing their knowledge of and adherence to the VB for VAP prevention.

### 3.4. Effect of the Participants’ Years of Experience on the Responses Expressing Their Knowledge of and Adherence to EBGs for VAP Prevention

Table 4 clarifies the effect of participants’ years of experience on the responses expressing their knowledge of and adherence to the VB for VAP prevention.

### 3.5. Effect of the Participants’ Job Titles on the Responses Expressing Their Knowledge of and Adherence to EBGs for VAP Prevention

Table 5 illuminates the effect of participants’ job titles on the responses expressing their knowledge of and adherence to the VB for VAP prevention.

### 3.6. Assessment of the Correct Knowledge of the Participants Regarding Evidence-Based VAP Prevention Guidelines According to Their Highest Educational Level

Table 6 presents an assessment of the participants’ correct knowledge of the EBGs for VAP prevention, categorized by their highest educational level.

### 3.7. Assessment of the Correct Knowledge of the Participants Regarding Evidence-Based VAP Prevention Guidelines According to Their Years of Experience

Table 7 presents an assessment of the participants’ correct knowledge of the EBGs for VAP prevention, categorized by their years of experience.

### 3.8. Assessment of the Correct Knowledge of the Participants Regarding Evidence-Based VAP Prevention Guidelines According to Their Job Title

Table 8 presents an assessment of the participants’ correct knowledge of the EBGs for VAP prevention, categorized by their job title.

### 3.9. Participants’ Self-Reported Barriers to Implementation of the Institutional VAP-Prevention Bundle

Table 9 clarifies the barriers reported by participants regarding causes of non-compliance with the VB. The primary implementation barrier preventing full compliance with the VB was identified as an educational deficit.

## 4. Discussion

Globally, VAEs represent a critical concern in ICUs with recent surveillance data demonstrating incidence rates ranging from 8.39 to 20.5 per 1000 ventilator-days across diverse healthcare settings [13,14]. The implementation of standardized VAE surveillance using CDC/NHSN criteria has revealed substantial variability in event detection and classification, with approximately 30% of mechanically ventilated ICU patients developing VAEs including VAP [15,16]. Recent evidence demonstrates that VAEs, including VAP, occurrence significantly impacts clinical outcomes, including increased ICU mortality with adjusted hazard ratios of 1.58 to 2.09, prolonged mechanical ventilation duration, and extended ICU length of stay [13,14]. Despite these adverse outcomes, compliance with prevention bundles remains suboptimal, with reported adherence rates varying from 43% to 69.2% among intensive care practitioners [17,18]. Studies conducted in Saudi Arabia and Middle Eastern contexts have identified critical barriers to effective implementation, including nursing staff shortages, inadequate training programs, limited resources, and insufficient enforcement of VAE prevention guidelines [2,18,19,20]. Beside the compliance with VB for VAP prevention, the integration of automated VAE surveillance systems has demonstrated feasibility and accuracy, with sensitivity reaching 98% and specificity of 100% in detecting VAE episodes [21,22]. However, significant gaps persist in healthcare practitioners’ knowledge of evidence-based prevention strategies [2,20]. International multicenter studies have emphasized the importance of continuous education, interdisciplinary collaboration, and resource optimization to enhance VB compliance and reduce VAEs incidence [15,16].

In this regard, this study provides essential baseline data to inform targeted interventions and policy enhancements within the Hail Region healthcare system, contributing to improved patient safety outcomes in mechanically ventilated patients. The present multi-centric investigation in Hail Region addresses critical knowledge gaps regarding the knowledge and adherence of ICU healthcare practitioners to the VB for VAP prevention. It also looked at the practitioners’ perceived barriers to effective VB deployment.

The demographic profile of the present study aligns with established patterns in Saudi and Middle Eastern critical care settings. The observed female predominance and age distribution are consistent with nursing workforce characteristics documented across Saudi tertiary hospitals [23,24]. For example, a descriptive cross-sectional study conducted by Barnawi et al. (2025) [25] at King Fahad General Hospital in Madinah, Saudi Arabia enrolled 96 ICU nurses and reported demographic similarities including female predominance (94.8%), bachelor’s-level education (77.1%), and age distribution showing 51% greater than 30 years with 39.6% between 26–30 years. However, their professional experience distribution differed, with 39.6% having more than 10 years compared to the current study’s 17.4%, and their training exposure was higher at 67.9% having received VAP prevention training compared to the current study’s 53.5%. The Madinah cohort demonstrated a 57.3% good knowledge level, indicating comparable educational needs across Saudi ICU settings despite geographic separation.

Likewise, multinational staffing reflects the broader dependence on expatriate healthcare professionals commonly seen across Gulf Cooperation Council countries [17,20]. The educational profile, with bachelor’s-level predominance, corresponds to contemporary nursing qualification standards in the region [26]. However, the experience distribution revealing a substantial proportion of early-career practitioners contrasts somewhat with the more experienced workforce reported in some Saudi ICU settings [27], though it aligns with global concerns about critical care workforce maturity [28].

The most critical finding in our study that nearly half of participants lacked formal VAP prevention training corroborates extensively documented educational gaps in Middle Eastern critical care contexts. This aligns disturbingly with Abdulrahman et al. (2024) findings that 81.6% of Saudi ICU nurses had not undergone specific VAP prevention training [23], and with reports identifying inadequate training and educational support as paramount barriers to guideline compliance across the Mediterranean region, Iran, Jordan, and Saudi Arabia [17,29]. In addition, an observational cross-sectional study by Jalal et al. (2022) [30] in eastern Saudi Arabia assessed 152 medical professionals including physicians, nurses, respiratory therapists, and interns, reporting that only 28.3% had received VAP prevention training within two years, which is substantially lower than our reported 53.5% training rates, and this training deficit correlated significantly with unsatisfactory performance.

Multiple studies have demonstrated that this educational deficit directly undermines compliance with evidence-based prevention protocols, regardless of nurses’ general clinical competence [29,31,32]. The training gap observed in Hail Region therefore represents not an isolated deficiency but rather a systemic regional challenge with well-documented implications for patient safety outcomes.

The high level of hand hygiene knowledge observed in our study aligns with the generally strong foundational understanding documented across Saudi critical care settings. The near-universal recognition of comprehensive hand hygiene requirements mirrors findings from multiple Saudi regions, where ICUs’ practitioners consistently demonstrate good-to-excellent knowledge of basic hand hygiene principles [33,34,35]. However, the persistent knowledge gap, though modest, reflects a pattern observed throughout Saudi healthcare facilities where complete comprehension remains elusive despite widespread educational emphasis [36,37].

The professional variation in hand hygiene knowledge with respiratory therapists demonstrating superior understanding compared to physicians in our study corroborates established patterns in Saudi critical care environments. Multiple studies have documented that nurses and allied health professionals consistently outperform physicians in hand hygiene compliance and knowledge assessments [38,39]. This professional hierarchy likely reflects the direct procedural responsibilities and intensive infection control training inherent to nursing and respiratory therapy practice, contrasting with physicians’ broader clinical focus [34,40].

The finding that experience level did not significantly influence hand hygiene knowledge contradicts some regional evidence suggesting that years of practice correlate positively with hand hygiene understanding and compliance [34,37]. Studies in Najran and Asir regions have demonstrated that older, more experienced nurses display superior hand hygiene knowledge and practices compared to their junior counterparts [34,41]. However, the present findings align with a recent systematic review by Alanazi et al. (2024) [35] indicating that when institutional hand hygiene education is robust and uniformly implemented, experience-related knowledge disparities diminish substantially. The absence of educational-level differences similarly reflects successful standardization of infection prevention curricula across nursing and medical education programs in Saudi Arabia, though this uniformity has not universally translated to complete knowledge attainment [42].

The high reported adherence to oral care protocols and chlorhexidine use in our study contrasts markedly with compliance patterns documented elsewhere in Saudi Arabia and the broader Middle East, where substantial implementation gaps persist despite widespread protocol availability [17,29]. This discrepancy may reflect social desirability bias in self-reported adherence measures or genuinely superior institutional support for oral care practices in the study facilities.

The knowledge gaps regarding chlorhexidine’s protective mechanism observed in our study align with growing clinical uncertainty reflected in recent systematic reviews and meta-analyses. While earlier evidence supported 0.12% chlorhexidine as effective for VAP prevention [43,44], contemporary evidence has generated increasingly contradictory findings. Multiple 2024 meta-analyses concluded that chlorhexidine demonstrates no significant advantage over placebo in preventing VAP or reducing mortality, ICU length of stay, or mechanical ventilation duration across any concentration [45,46]. Some investigators have even raised concerns about potential adverse effects, including increased mortality risk and ARDS from aspiration [47,48]. This evolving evidence base may partially explain why 31.4% of our practitioners in the present study demonstrated incomplete or incorrect understanding of chlorhexidine’s effects, the literature itself remains contentious.

The educational-level differences in chlorhexidine knowledge, with master’s degree holders demonstrating superior understanding, corroborate established patterns linking advanced education to evidence-based practice integration [49,50]. However, the finding that experience significantly influenced both knowledge and adherence contradicts some regional evidence suggesting that formal education outweighs clinical experience in determining evidence-based practice adoption [17,51]. The experience-dependent gradient observed in this study more closely aligns with the critical care literature demonstrating that novice practitioners require extended clinical exposure before effectively translating theoretical knowledge into consistent protocol adherence [40]. This pattern underscores persistent deficiencies in orientation programs for newly hired ICU staff, a challenge documented extensively across Saudi healthcare institutions where onboarding processes often inadequately prepare novices for complex infection prevention requirements [2,29].

The universal practical compliance with HOB elevation reported in our study contrasts dramatically with documented implementation patterns across international critical care settings, where adherence to HOB elevation goals remains persistently problematic. Studies consistently report that HOB elevation to ≥30° is achieved in only 27.8% to 38.5% of measurements, with many patients experiencing entire 24-h periods without achieving the elevation target [52,53,54]. This substantial discrepancy between the present findings and broader literature likely reflects either social desirability bias in self-reported compliance measures or genuinely superior institutional culture regarding positioning protocols in the study facilities.

On the other hand, the inverse educational gradient observed in our study, where diploma and bachelor’s-prepared practitioners demonstrated superior knowledge compared to master’s and doctorate holders, aligns with documented patterns in bedside nursing competencies. Multiple studies corroborate that advanced degree programs emphasize research methodology, leadership, and theoretical frameworks rather than granular clinical procedures, creating knowledge gaps in fundamental nursing interventions among highly educated practitioners [50,55]. The professional hierarchy in positioning knowledge, with nurses demonstrating the highest accuracy despite physicians showing the best understanding of positioning rationale, reflects the well-documented disconnect between knowledge authority and practice responsibility characteristic of hierarchical healthcare teams [2,27].

Regarding the optimal elevation angle itself, current evidence presents evolving complexity beyond the traditional 30–45° recommendation. While earlier guidelines uniformly recommended 30–45° elevation [56,57], recent trials demonstrate that 45° elevation significantly reduces VAP incidence compared to lower angles but simultaneously increases pressure ulcer risk [58,59,60]. This trade-off has prompted expert panels to acknowledge scientific uncertainty and recommend 30–45° positioning only “as long as it does not pose risks or conflicts with other nursing tasks, medical interventions or patients’ wishes” [56,61]. The knowledge gaps observed in this study regarding specific elevation angles therefore mirror genuine clinical equipoise within the critical care community about optimal positioning strategies.

The finding that positioning knowledge showed no experience-related variation contradicts extensive evidence demonstrating that novice practitioners struggle with multiple competing priorities and often deprioritize seemingly simple interventions like positioning maintenance [52,62]. It was reported that nurse workload is the main barrier to maintaining adherence to HOB elevation, and compliance worsens especially during busy shifts, irrespective of nurses’ knowledge levels [52]. The lack of experience-related effects in the present study may reflect either ceiling effects in knowledge assessment or inadequate capture of the knowledge-practice gap that characterizes positioning interventions across different experience levels.

The strong knowledge base regarding daily SBT frequency observed in our experienced practitioners aligns perfectly with current EBGs. The 2024 American Association for Respiratory Care Clinical Practice Guideline explicitly recommends “a standardized approach to assessment and, if appropriate, completion of an SBT before noon each day” (conditional recommendation, very low certainty) [63,64]. This daily frequency recommendation reflects consensus that once reversible causes for SBT failure are corrected, subsequent trials should occur every 24 h rather than more frequently [63,65]. It was reported that comparing once-daily versus more frequent screening showed no significant difference in time to successful extubation [65,66], supporting the daily approach used by the experienced practitioners in this study.

The substantial performance gap among novice practitioners observed in this study corroborates extensively documented patterns in the critical care weaning literature. It was reported that newly graduated or recently hired ICU staff struggle disproportionately with complex weaning assessments, often delaying liberation from mechanical ventilation due to inadequate clinical judgment regarding readiness criteria [54]. This phenomenon reflects the multifaceted nature of SBT protocols, which require integration of respiratory mechanics understanding, hemodynamic assessment, neurological evaluation, and continuous patient monitoring skills that are developed only through supervised clinical experience [63,67]. The finding that educational level did not influence SBT knowledge contradicts some evidence suggesting advanced degrees enhance protocol comprehension but aligns with research indicating that weaning competency depends primarily on bedside exposure rather than theoretical education [50,68].

The high reported adherence to SBT performance represents an encouraging finding but must be interpreted cautiously given substantial international evidence of poor SBT implementation. It was reported that delays in identifying weaning readiness are a persistent issue across ICU settings. Landmark studies have found that only about 75% of patients succeed in their first SBT when it is eventually attempted, suggesting that clinicians consistently postpone trials beyond the optimal timing. [63,64]. Furthermore, respiratory therapist-driven protocols with daily screening improve liberation times compared to physician-directed approaches, emphasizing the importance of interprofessional collaboration in SBT implementation that transcends individual professional categories [63]. The experience-dependent performance patterns observed in this study underscore the critical need for enhanced preceptorship programs pairing novice practitioners with experienced mentors during the initial months of ICU practice, a strategy demonstrated to accelerate competency development in complex weaning decisions [2,54].

The high reported adherence to sedation depth monitoring and daily sedation interruption in our study contrasts markedly with documented compliance patterns in international critical care settings. Despite widespread recommendations for daily spontaneous awakening trials (SATs) since the landmark 1996 Ely et al. trial [69], many studies consistently demonstrate suboptimal implementation globally even in academic centers [70,71,72,73]. The 2025 updated Society of Critical Care Medicine Clinical Practice Guidelines continue to conditionally recommend daily awakening trials in addition to targeting light sedation, yet compliance has not returned to pre-COVID levels despite these persistent recommendations [70]. This discrepancy between the present findings and broader literature likely reflects either social desirability bias in self-reported measures or genuinely superior institutional sedation culture in the study facilities.

The absence of educational-level differences in sedation practices aligns with evidence suggesting that sedation protocol adherence depends more heavily on institutional culture and nursing autonomy than individual educational credentials [74,75]. Multiple studies demonstrate that nurse-led sedation protocols with explicit authority to interrupt sedation without physician permission achieve superior outcomes compared to physician-directed approaches, regardless of nurses’ degree levels [76,77]. This emphasizes the critical importance of protocol design that empowers bedside practitioners with clear decision-making authority.

The profound experience-dependent gap in sedation interruption practice particularly the 44.44% deficit among novice practitioners in our study corroborates extensively documented patterns in the critical care sedation management literature. Multiple international studies identify that newly hired ICU staff demonstrate substantially lower adherence to sedation protocols due to discomfort managing agitated patients, inadequate understanding of sedation pharmacokinetics, fear of adverse events during awakening, and unclear institutional support for protocol implementation without direct physician oversight [54,78]. This phenomenon carries profound clinical consequences, as failure to perform daily sedation interruption directly prolongs mechanical ventilation duration by 1–2 days, increases ICU length of stay by similar margins, elevates delirium incidence, and contributes to long-term cognitive dysfunction and ICU-acquired weakness [79,80,81].

Protocolized sedation with daily interruption has demonstrated consistent mortality reduction (RR 0.80, 95% CI 0.68–0.93) and decreased ventilation days (mean difference −1.12 days) in meta-analyses, making the performance gap among novices a patient safety imperative requiring urgent intervention [82,83]. The universal compliance among experienced practitioners demonstrates that sedation interruption protocols are feasible and sustainable once practitioners develop necessary competencies, but the novice deficit necessitates structured preceptorship pairing new hires with experienced mentors, explicit protocol authority documentation, simulation-based training in managing aroused patients, and mandatory competency verification before independent sedation management authorization [2,70].

The major knowledge gaps about subglottic secretion drainage (SSD) identified in our study are especially troubling, considering the strong body of evidence supporting this technology. Multiple studies consistently demonstrate that SSD reduces VAP incidence [84,85,86]. Remarkably, a 2020 landmark meta-analysis became the first to demonstrate mortality reduction associated with SSD use (RR 0.88, 95% CI 0.80–0.97), elevating this intervention beyond infection prevention to survival benefit [84]. Technology demonstrates efficacy for early-onset VAP, with risk reduction reaching 76% (RR 0.24, 95% CI 0.06–0.84) when implemented within the first 5–7 days of mechanical ventilation [87].

The disturbing finding that 55.56% of novice practitioners incorrectly believed SSD tubes increase VAP risk represents a critical misconception that directly undermines evidence-based practice. This reversal of understanding contradicts all published research, which uniformly reports protective effects without identifying mechanisms by which SSD could increase infection risk [84,85,86,88]. The misconception may stem from confusion regarding potential adverse effects, specifically tracheal mucosa injury documented with continuous high-pressure suction though studies demonstrate that intermittent low-pressure drainage minimizes tissue trauma while preserving efficacy [89]. This knowledge deficit among novices likely contributes to the documented underutilization of SSD technology across ICU settings despite strong guideline recommendations [85,88].

The educational gradient observed in our study with master’s degree holders demonstrating perfect understanding while diploma holders showed zero correct responses aligns with the technical complexity of understanding device mechanisms and interpreting meta-analytic evidence. However, this pattern contradicts the fundamental principle that frontline bedside practitioners should possess working knowledge of equipment they manage daily [2,90]. The finding that even experienced practitioners demonstrated only 70–84% correct understanding reveals system-wide educational deficiencies regarding this cost-effective intervention that prevents 20 VAP cases per 500 ventilated patients, reduces ICU occupancy by 86 bed-days annually, and generates healthcare savings despite initial equipment costs [87,91].

Despite the evidence supporting SSD use, international surveys consistently document that this technology remains underused in clinical practice due to initial equipment costs, variable secretion volumes between patients, doubts about safety, and institutional purchasing barriers [85,88,92]. Current guidelines conditionally recommend SSD for patients expected to require mechanical ventilation exceeding 48–72 h, yet implementation rates remain suboptimal even in resource-rich settings [85,87]. Accordingly, the knowledge shortfalls seen in this study point to wider systemic problems in translating strong evidence into frontline training and institutional procurement policies.

The substantial uncertainty regarding kinetic bed therapy observed in our study reflects genuine ongoing controversy within the critical care evidence base itself. While a landmark 2006 meta-analysis of 15 trials demonstrated that kinetic beds significantly reduce nosocomial pneumonia odds (pooled OR 0.38, 95% CI 0.28–0.53), this apparent benefit came with a critical caveat: no trial met all validity criteria, and more importantly, kinetic therapy showed no reduction in mortality (pooled OR 0.96, 95% CI 0.66–1.14), duration of mechanical ventilation, ICU length of stay, or hospital length of stay [93]. This unexpected finding, less pneumonia without better overall clinical outcomes, has fueled continued skepticism about whether the pneumonia reduction reflects genuine prevention or is instead a diagnostic artifact caused by improved pulmonary mechanics that change how chest radiographs are interpreted [94].

The experience-dependent knowledge pattern observed in this study aligns with clinical reality that kinetic beds remain specialized, expensive equipment not universally available across ICU settings. Technology assessments by health authorities consistently conclude that while kinetic beds may reduce pneumonia odds, the poor methodological quality of supporting trials, lack of mortality benefit, and substantial resource costs prevent definitive recommendations for routine implementation [93,95,96]. EBGs have progressively deemphasized kinetic therapy compared to earlier recommendations, with the SHEA/IDSA/APIC compendium mentioning it only peripherally [7] and the ISID guidelines prioritizing early mobilization programs over passive rotation technology [61].

The finding that even our master’s and doctorate holders demonstrated only 77–85% correct understanding contradicts the assumption that advanced education ensures knowledge of all VAP prevention modalities. This pattern more accurately reflects rational resource allocation decisions by healthcare institutions that have deprioritized kinetic bed acquisition given the ambiguous benefit-to-cost ratio [93,97]. It was revealed that the VBs with the greatest VAP reductions integrate HOB elevation, oral hygiene, SSD, and sedation regimens, none of which require costly specialized beds, yielding declines of more than 65% when these components are implemented correctly [98,99].

The knowledge gaps regarding kinetic therapy therefore represent not simply educational deficiencies but rather appropriate clinical agnosticism toward a technology whose impressive pneumonia reduction in pooled analyses has failed to translate into the mortality improvements that matter most to patient-centered outcomes [54,93]. The modest enthusiasm for kinetic beds reported in our study appropriately reflects current evidence uncertainty rather than problematic knowledge deficits requiring urgent remediation until methodologically rigorous trials demonstrate survival benefit, ICU and hospital length of stay reductions, or cost-effectiveness justifying capital expenditure.

The finding that only 67.4% of our practitioners correctly identified oral intubation as preferred reflects concerning knowledge gaps regarding a well-established VAP prevention principle. Major EBGs consistently recommend oral over nasal intubation as a fundamental component of VB, with this recommendation appearing in the CDC/HICPAC guidelines [100], SHEA/IDSA/APIC compendium [7], and ISID international guidelines [61] based on strong evidence that nasal intubation increases sinusitis risk and subsequent VAP development. Approximately one-third of practitioners demonstrating misconceptions or uncertainty represents a modifiable knowledge deficit with direct clinical implications, as intubation route decisions occur at the critical point of airway establishment and cannot be retrospectively corrected without reintubation.

The paradoxical finding that our doctorate holders demonstrated lower accuracy (28.57%) compared to diploma holders (100%) aligns with patterns observed throughout this study where advanced research-focused degrees do not guarantee superior knowledge of bedside clinical practices. This educational gradient contradicts the assumption that higher academic credentials translate uniformly to comprehensive clinical knowledge, instead reflecting the specialized nature of doctoral training that emphasizes research methodology and theoretical frameworks over procedural decision-making. The pattern corroborates established learning theory distinguishing between academic knowledge and procedural competence, where the latter develops primarily through direct clinical exposure rather than didactic education [68].

The absence of significant experience-related differences in intubation route knowledge contrasts with the experience-dependent patterns observed for other VAP prevention interventions in this study. This finding suggests that intubation route selection receives inadequate emphasis throughout professional careers, failing to improve substantially even with accumulated clinical exposure [54]. Multiple VAP prevention research identifies persistent deficits in understanding fundamental airway management principles across experience levels, particularly when specific interventions fall outside practitioners’ direct procedural responsibilities [54,101].

The significant professional category differences observed likely reflect the physician-driven nature of intubation decisions, with physicians and respiratory therapists demonstrating superior knowledge compared to nurses who typically do not perform intubations but must understand the rationale for equipment selection [2,27]. However, the substantial knowledge gaps across all professional groups underscore systemic educational deficiencies regarding this EBG, suggesting that even professionals directly responsible for intubation may harbor misconceptions about VAP risk modification through route selection [54,101]. This pattern emphasizes the need for interprofessional VAP prevention education that ensures all team members regardless of their procedural role understand the infection prevention rationale underlying airway management decisions made at the point of intubation.

The universal self-reported DVT prophylaxis compliance rate observed in our study contrasts dramatically with documented adherence patterns across international critical care settings, where prophylaxis implementation remains persistently suboptimal despite clear guidelines. Large-scale database analyses reveal that only 85.6% of ICU patients receive pharmacologic venous thromboembolism (VTE) prophylaxis, with multinational studies demonstrating that merely 58% of surgical patients and 37% of medical patients receive recommended prophylaxis [102,103]. Indian tertiary care audits report baseline compliance rates of 69.9% before quality improvement interventions, rising to 88.4% only after structured protocol implementation [104]. This substantial discrepancy between the present findings and broader literature strongly suggests social desirability bias in self-reported measures, where practitioners recognize DVT prophylaxis as an unambiguous standard of care and therefore report perfect adherence regardless of actual practice patterns [54].

The clinical significance of this implementation gap cannot be overstated, as missed VTE prophylaxis directly increases mortality. Critically ill patients who fail to receive indicated prophylaxis experience 65% higher adjusted odds of in-hospital death (OR 0.35, 95% CI 0.34–0.37), despite paradoxically shorter ICU lengths of stay, suggesting that prophylaxis omission identifies a higher-acuity population at elevated mortality risk [105]. This mortality association persists even after adjustment for severity scores, emphasizing that DVT prophylaxis represents not merely infection prevention but fundamental life-saving intervention [102].

The absence of variation across educational levels, experience strata, and professional categories observed in our study aligns with a small subset of exemplary institutions achieving near-universal compliance through systematic implementation strategies. A 2025 Indian tertiary care audit demonstrated 100% compliance with DVT risk assessment documentation and contraindication recording, attributing this success to structured protocols, mandatory electronic risk assessment tools, and periodic audits with feedback [104]. International consensus guidelines explicitly recommend that passive education alone proves ineffective for improving thromboprophylaxis adherence, instead requiring active interventions such as computerized decision support systems, properly designed order sheets, and systematic audits with outcome transparency [106,107].

If the reported universal compliance in this study reflects genuine practice rather than reporting bias, it represents a model implementation worthy of detailed investigation and replication. The factors potentially enabling such success include simplified protocols eliminating clinical judgment requirements, institutional culture prioritizing thromboprophylaxis as a quality metric, automated electronic alerts triggering prophylaxis orders, pharmaceutical protocols permitting nurse-initiated prophylaxis without physician orders, and systematic audit-and-feedback cycles with visible compliance dashboards [104,106,108]. Understanding and replicating these implementations strategies could address the persistent compliance gaps documented for more complex VAE prevention interventions such as sedation interruption, SBTs, and oral care protocols where clinical judgment, patient cooperation, and procedural complexity create barriers to universal adherence [54,70].

The near-universal self-reported compliance with stress ulcer prophylaxis (SUP) observed in our study contrasts sharply with documented patterns of inappropriate overuse rather than underuse across international critical care settings. Multiple recent studies reveal that while prophylaxis administration rates remain high, appropriateness of use according to evidence-based guidelines remains concerningly low, with 66–69% of SUP prescriptions lacking appropriate indications based on American society of health-system pharmacists (ASHP) and Portuguese Society of Intensive Care criteria [109,110,111]. A 2025 Saudi Arabian cross-sectional study found that among 274 hospitalized patients receiving PPIs for SUP, only 31.02% had appropriate indications, while 68.98% received prophylaxis without meeting guideline criteria [110]. This pattern suggests that the clinical challenge with SUP lies not in achieving high prescription rates but rather in ensuring selective, guideline-directed use that balances bleeding prevention against potential adverse effects [109,112].

Contemporary evidence has prompted fundamental reevaluation of universal SUP application versus selective use in genuinely high-risk patients. It was emphasized that only patients with specific risk factors, including mechanical ventilation exceeding 48 h, coagulopathy, history of gastrointestinal ulceration, or major burns, derive clear benefit from pharmacological prophylaxis [113,114]. Modern ICU practice with early enteral nutrition, improved hemodynamic management, and reduced illness severity has decreased baseline bleeding risk to approximately 3–4% without prophylaxis, far lower than the 5–25% rates documented in historical studies that established SUP as standard practice [112,113,115]. Some experts now argue that tolerance of enteral nutrition serves as a surrogate marker for adequate splanchnic perfusion, potentially obviating prophylaxis necessity in patients without additional risk factors [112,114].

The contrast between near-perfect adherence to passive pharmaceutical interventions (DVT prophylaxis and SUP both approaching 100%) versus more variable compliance with active procedural interventions (sedation interruption, SBTs, oral care at 95.3%) observed in our study corroborates established implementation science principles. Medication orders requiring single initial prescription followed by automated continuation achieve higher sustained compliance than interventions requiring repeated daily reassessment, clinical judgment, patient cooperation, and time-intensive procedures [54]. This compliance hierarchy indicates that quality improvement resources should prioritize high-effort interventions with documented adherence gaps rather than pharmaceutical interventions already achieving near-universal implementation [54,109].

However, the appropriateness question remains paramount: achieving 98.8% SUP compliance may represent problematic overuse rather than quality achievement if prescriptions lack proper indications. A study implementing clinical pharmacist-guided protocols demonstrates that appropriate indication rates can improve from 38.3% to 47.8% through education, with corresponding cost savings of $26–34 per patient by eliminating inappropriate prescriptions [116]. Furthermore, inappropriate SUP use increases risks of VAP, *Clostridium difficile* infection, and medication costs without conferring bleeding prevention benefit [110,111,117]. The universal compliance observed therefore warrants verification not only of whether SUP is administered but whether each prescription meets evidence-based indication criteria, transforming the metric from simple compliance to appropriate prescribing.

The variable understanding of VB composition observed in our study reflects documented inconsistencies in bundle definitions across international guidelines and institutions. A systematic review of 36 studies involving 116,873 mechanically ventilated patients revealed substantial heterogeneity in bundle components, with HOB elevation being the most universally included element (*n* = 83,146 participants), followed by oral care (*n* = 80,787), but with considerable variation in other components [118]. The IHI recommended a VB to reduce morbidity and death in VAP patients [8]. Improving patient safety and lowering problems related to mechanical ventilation in the three central hospitals included in our study depend on implementing the IHI strategies, yet international guidelines vary substantially in their recommendations [119,120]. The finding that 81.4% correctly identified antibiotic nebulization as non-standard aligns with EBGs that explicitly do not recommend routine aerosolized antibiotics for VAP prevention due to lack of efficacy and concerns about antimicrobial resistance [61]. However, the misconceptions where practitioners incorrectly identified legitimate bundle elements as non-standard, particularly DVT prophylaxis (8.1%) and HOB elevation (4.7%), reflect genuine confusion stemming from inconsistent bundle definitions across institutions and evolving guidelines [57].

The dramatic experience-dependent gradient, where 77.8% of novice practitioners misidentified core VB components compared to only 8–12% of experienced practitioners, corroborates international knowledge assessments. A Nepalese study found that 51.5% of critical care nurses had poor knowledge of VAP prevention bundles, with only 3% demonstrating good knowledge, and significant associations between knowledge levels and clinical experience [121]. Similarly, studies across multiple countries document that newly hired ICU staff demonstrate substantially inferior understanding of evidence-based VAP prevention interventions compared to experienced colleagues [54,101].

The professional hierarchy in bundle knowledge, with respiratory therapists achieving 100% accuracy, nurses 87.7%, but physicians only 50%, represents a particularly concerning finding with direct clinical implications. This pattern contradicts the expectation that physicians, who typically prescribe and authorize bundle interventions, should demonstrate superior understanding of evidence-based components [2]. It was documented that physician knowledge deficits create significant barriers to nursing implementation efforts, such as incomplete bundle orders or failure to support nursing-initiated interventions directly undermine prevention fidelity [7]. The physician knowledge gap observed in this study aligns with broader evidence that medical training inadequately emphasizes infection prevention protocols compared to nursing and allied health curricula, creating interprofessional knowledge asymmetries that compromise patient safety [54].

The finding that only 57% of our sample correctly identified factors that do not increase VAP risk reveals substantial knowledge deficits regarding VAP pathophysiology and epidemiology. The misconception among 14% of practitioners that prolonged mechanical ventilation does not increase VAP risk contradicts overwhelming evidence from various research consistently identifying duration of mechanical ventilation as one of the most robust and dose-dependent risk factors for VAP development [122,123,124].

The concerning result is that 27.9% of our practitioners incorrectly believe that reducing clinical staffing does not increase the risk of VAP, which directly contradicts established infection prevention research. Adequate nurse-to-patient ratios are essential for performing labor-intensive preventive interventions including oral care, patient repositioning, endotracheal suctioning, and respiratory assessments [2,54,101]. Studies consistently demonstrate that understaffing compromises adherence to VBs, as time-intensive procedures are deprioritized when workload exceeds capacity [54,118]. The knowledge gap regarding staffing’s impact on infection risk suggests inadequate understanding of how system-level factors influence individual patient outcomes.

The experience-dependent pattern, where 55.56% of novice practitioners misidentified prolonged mechanical ventilation as safe, aligns with documented patterns of inadequate pathophysiology education among newly hired ICU staff [54,101,121]. This fundamental misunderstanding of the dose–response relationship between ventilator exposure duration and cumulative infection risk reflects insufficient emphasis on mechanistic understanding during orientation programs [54]. Contemporary VAP education research emphasizes that teaching not only what to do (bundle elements) but why interventions work (underlying pathophysiology) builds conceptual frameworks supporting appropriate clinical reasoning and sustained protocol adherence [101,121].

Current evidence identifies multiple validated VAP risk factors beyond prolonged ventilation, including male gender (OR 1.50), reintubation, enteral feeding, impaired consciousness, nasogastric tube use, H2-blocker administration, tracheostomy, prior antibiotics, neuromuscular blockers, and higher APACHE II scores [122,123,124]. Conversely, intermittent sedation strategies and antibiotic prophylaxis demonstrate protective effects [54,123]. The knowledge deficits observed in this study regarding risk factor identification suggest that educational interventions should adopt integrated approaches combining pathophysiology instruction, epidemiological evidence, and clinical decision-making scenarios that require practitioners to apply risk assessment knowledge to actual patient care situations rather than simply memorizing lists of risk factors [121].

The self-reported 93% VB compliance observed in our study substantially exceeds documented adherence rates across international critical care settings. A 2025 multicenter study in Oman revealed that nurses’ actual compliance with VAP guidelines reached only 69%, with compliance decreasing progressively over patients’ admission periods [120]. Similarly, a 2025 Saudi Arabian multicenter investigation documented that merely 43% of intensive care nurses demonstrated high compliance with VB, with major barriers including nursing staff shortages, inadequate training, limited resources, and insufficient institutional support [27]. Most strikingly, a Vietnamese longitudinal surveillance study over 24 months revealed complete VB compliance in only 1.8% of assessments despite average individual component adherence of 84.1% [125], and an Indonesian tertiary hospital reported just 5% complete bundle compliance [126]. These patterns strongly suggest that the high compliance reported in the present study reflects social desirability bias inherent in self-reported measures rather than actual practice patterns [54,120,127].

The experience-dependent compliance gap among our novice practitioners with only 66.67% reporting bundle compliance compared to 96–100% among our experienced staff aligns perfectly with documented patterns across multiple countries. Studies consistently demonstrate that newly hired ICU staff show substantially lower adherence to complex multi-component bundles due to inadequate orientation, insufficient procedural confidence, time management difficulties, and incomplete institutional culture integration [25,54]. A 2025 Ethiopian cross-sectional study found that less experienced nurses demonstrated significantly lower compliance despite adequate theoretical knowledge, emphasizing that bundle implementation requires procedural skills, clinical judgment, and workflow integration that develop only through supervised practice [128]. The variations in guidelines-related responses between physicians and nurses highlight the significance of professional roles, education, and interdisciplinary communications [129].

This 33.33% non-compliance gap among novices represents substantial patient safety risk, as studies demonstrate that mean compliance was significantly higher in non-VAP patients (72.9%) compared to those who developed VAP (56.6%), with compliance linked to shorter length of stay, fewer mechanical ventilation days, and reduced hospital costs [120].

The identification of educational deficits as the predominant barrier aligns with systematic reviews identifying knowledge gaps as the most frequently reported impediment to VAP prevention across diverse settings [118]. However, this represents a highly modifiable barrier amenable to structured interventions. Multiple studies demonstrate that comprehensive educational programs combining didactic instruction, simulation training, and bedside mentorship can improve bundle compliance from baseline rates of 70–84% to sustained rates exceeding 95–97%; which is the threshold associated with near-zero VAP incidence [130,131,132]. An Indian tertiary care hospital achieved compliance improvement from 90% in 2021 to 97% in 2022 through systematic quality improvement initiatives, resulting in VAP rate reductions exceeding 70% [131]. The extra barriers of limited resources (15%) and disagreement with the evidence (20%) call for different strategies: allocating capital budgets for equipment-intensive interventions and using journal clubs with critical appraisal training to address skepticism about the evidence [98,118].

Critically, achieving greater than 95% compliance with VB represents the threshold necessary to approach zero VAP incidence, yet few institutions sustain such adherence without complex multifaceted interventions including automated surveillance, real-time feedback systems using electronic dashboards, leadership engagement, and continuous audit cycles with immediate resolution of non-conformities [118,133]. Facilities achieving VAE rates below 2 per 1000 ventilator-days typically demonstrate bundle compliance exceeding 95%, systematic staff education programs, and real-time surveillance with immediate feedback—elements requiring validation in facilities reporting exceptional performance [98,125].

Several factors, including the frequent use of invasive procedures and devices, immunosuppression, pre-existing comorbidities, fragility, and advanced age, put patients in ICUs at high risk for HAIs [134]. Assessing ICUs’ practitioners’ knowledge and adherence to VB is therefore essential as they are in the front lines for keeping patients alive. The correlation between practitioner knowledge deficits, bundle compliance gaps, and perceived barriers observed across our facilities corroborates established quality improvement science demonstrating direct relationships between educational interventions, protocol adherence, and infection outcomes [27]. The present study’s identification of modifiable barriers; particularly educational deficits, provides actionable targets for quality improvement initiatives that could bridge the compliance gap, especially among novice practitioners who demonstrate the most substantial adherence deficits and represent future ICU workforce sustainability.

Finally, when applying our study findings more broadly, it is essential to consider several limitations. First, the use of self-reported data collected via cross-sectional surveys, and convenient recruitment may introduce social desirability bias, leading practitioners to overstate their knowledge and adherence to the VB. This could conceal real compliance gaps and reduce the accuracy of associations between knowledge and actual adherence. Second, the relatively small sample size (*n* = 86 practitioners) limits statistical power to detect differences across professional categories and restricts subgroup analyses, especially for smaller groups such as respiratory therapists (12.8%), which may result in underestimating effect sizes. Third, because the cross-sectional survey design measures knowledge and compliance at a single point in time without longitudinal follow-up, it cannot assess knowledge retention, the durability of practice, the effect of training on compliance, or how identified barriers may evolve. Fourth, this study was performed on a regional cohort from the northern part of Saudi Arabia, which may restrict the generalizability of the findings to other Saudi regions or healthcare systems. Furthermore, the questionnaire was not validated, thereby limiting its robustness and reproducibility. However, it was adapted from the validated questionnaire developed by Paliwal et al. [12]. This tool had previously undergone expert review and was specifically designed to evaluate both knowledge and compliance related to the ventilator bundle in the ICU settings. Moreover, the study team assessed the questionnaire’s content and format, and a statistician revised its statistical design before it was distributed. Future research should build on this work using interdisciplinary comparisons, qualitative interviews, and multicenter national surveys.

## 5. Conclusions and Recommendations

This comprehensive study reveals substantial heterogeneity in VB knowledge and adherence across educational levels, experience strata, and professional categories within the Hail Region healthcare system. The findings indicate that while some fundamental interventions achieve excellent compliance (hand hygiene knowledge, head elevation practice, DVT prophylaxis), critical gaps persist in complex interventions requiring clinical judgment and interdisciplinary coordination, particularly among novice practitioners. The dramatic experience-dependent gradients in sedation interruption, SBTs, and VB compliance underscore urgent need for enhanced orientation, structured mentorship, and competency verification systems targeting newly hired ICU staff. Professional category differences, particularly physicians’ lower accuracy regarding VB components despite their role as protocol prescribers, indicate need for interdisciplinary education ensuring consistent understanding across all team members. The identification of educational deficit as the predominant implementation barrier suggests that systematic education programs addressing documented knowledge gaps represent the most actionable target for immediate quality improvement within the Hail Region critical care system.

It is recommended to implement mandatory, competency-based VB training programs for all ICU practitioners across Hail Region hospitals, with particular emphasis on novice practitioners (less than one year experience) and physician education to address identified knowledge gaps in bundle components, sedation interruption, and SBT protocols. Furthermore, conducting larger, multicenter, prospective interventional studies with pre- and post-implementation assessments to robustly validate the impact of structured educational interventions on knowledge retention, VB adherence rates, and VAP incidence reduction, with longitudinal follow-up to evaluate sustainability of practice changes over 12–24 months. Moreover, undertaking direct comparative studies correlating objective bundle compliance monitoring (through direct observation audits and automated surveillance systems) versus self-reported adherence to quantify the magnitude of social desirability bias and establish accurate baseline compliance rates necessary for meaningful quality improvement initiatives.

## Figures and Tables

**Table 1:** Demographic data of the participants.

Participants’ Demographics		Total (*n* = 86)	
		*n*	%
Age	20–30 years	22	25.6%
	31–40 years	45	52.3%
	41–50 years	17	19.8%
	More than 50 years	2	2.3%
Gender	Male	36	41.9%
	Female	50	58.1%
Nationality	Saudi	35	40.7%
	Non-Saudi	51	59.3%
Job title	Physician	18	20.9%
	Nurse	57	66.3%
	Respiratory therapist	11	12.8%
Educational level	Diploma	1	1.2%
	Bachelor’s degree	69	80.2%
	Master’s degree	9	10.5%
	Doctorate	7	8.1%
Years of experience in ICU	Less than 1 year	9	10.5%
	1–3 years	17	19.8%
	4–6 years	25	29.1%
	7–9 years	20	23.3%
	More than 10 years	15	17.4%
Training or seminars regarding EBGs for VAP prevention	Yes	46	53.5%
	No	40	45.3%

ICU: intensive care unit, VAP: ventilator-associated pneumonia, EBGs: evidence-based guidelines.

**Table 2:** Total participants’ responses regarding knowledge of and adherence to EBGs for VAP prevention.

Questions	Participants’ Responses	Total Responses (*n* = 86)	
		*n*	%
**The first section; knowledge of EBGs for VAP prevention**			
Q1: Oral vs. nasal route for endotracheal intubation	Oral intubation is preferred over nasal route for VAP prevention	58	67.4%
	Nasal intubation is preferred over oral route for VAP prevention	9	10.5%
	No difference in VAP incidence between both route	13	15.1%
	I don’t know	6	7%
Q2: Which of the following is not a standard component of the VB?	Head-end elevation	4	4.7%
	Peptic ulcer prophylaxis	2	2.3%
	DVT prophylaxis	7	8.1%
	Nebulization with antibiotic	70	81.4%
	Daily sedation vacation and assessment of readiness to extubate	3	3.5%
Q3: Which of the following does not increase the incidence of VAP?	Intermittent sedation	49	57%
	Aspiration of oropharyngeal and gastric secretion	1	1.2%
	Reduce clinical staffing	24	27.9%
	Prolonged mechanical ventilation	12	14%
Q4: To reduce the risk of VAP, the head end of the bed should be elevated to:	5–10°	1	1.2%
	10–20°	2	2.3%
	20–30°	5	5.8%
	30–45°	78	90.7%
Q5: How often should you attempt awakening and spontaneous breathing trials?	Every 6 h	3	3.5%
	Every 12 h	3	3.5%
	Daily	79	91.9%
	Never	1	1.2%
Q6: How often should you perform hand hygiene?	Before entering the ICU	0	0.0%
	Before and after contact with the ventilator or ventilator circuit	1	1.2%
	Before and after touching the patient	7	8.1%
	All the above	78	90.7%
Q7: Endotracheal tubes with extra lumen for drainage of subglottic secretions	These endotracheal tubes reduce the risk of VAP	62	72.1%
	These endotracheal tubes increase the risk of VAP	14	16.3%
	These endotracheal tubes don’t influence the risk of VAP	3	3.5%
	I don’t know	7	8.1%
Q8: Kinetic vs. standard beds	Kinetic beds increase the risk of VAP	6	7%
	Kinetic beds reduce the risk of VAP	51	59.3%
	The use of kinetic beds doesn’t influence the risk of VAP	12	14%
	I don’t know	17	19.8%
Q9: Patient positioning	Supine positioning is preferred for VAP prevention	5	5.8%
	Semi-recumbent positioning is preferred for VAP prevention	69	80.2%
	The position of the patient doesn’t influence the risk of VAP	8	9.3%
	I don’t know	4	4.7%
Q10: Usage of 0.12% chlorhexidine gluconate antiseptic oral rinse	0.12% chlorhexidine oral rinse reduces the risk of VAP	59	68.6%
	0.12% chlorhexidine oral rinse increase sthe risk of VAP	7	8.1%
	0.12% chlorhexidine oral rinse doesn’t influence the risk of VAP	12	14%
	I don’t know	8	9.3%
**The Second section; Adherence to the VB in daily practice**			
Q1: I always comply with the Institutional VB.	Yes	80	93%
	No	6	7%
Q2: I adhere to existing oral care protocol.	Yes	82	95.3%
	No	4	4.7%
Q3: I always use chlorhexidine oral rinse as recommended.	Yes	70	81.4%
	No	16	18.6%
Q4: I assess the depth of sedation as often as recommended.	Yes	84	97.7%
	No	2	2.3%
Q5: I interrupt continuous sedative infusions as recommended.	Yes	82	95.3%
	No	4	4.7%
Q6: I perform spontaneous breathing test as recommended.	Yes	82	95.3%
	No	4	4.7%
Q7: I always keep head of bed elevated to 30–45°.	Yes	86	100%
	No	0	0%
Q8: I always make sure that mechanical DVT prophylaxis is inserted as recommended.	Yes	86	100%
	No	0	0%
Q9: I always give pharmacological DVT prophylaxis as recommended.	Yes	86	100%
	No	0	0%
Q10: I always give pharmacological peptic ulcer prophylaxis as recommended.	Yes	85	98.8
	No	1	1.2

VAP: ventilator-associated pneumonia, VB: ventilator bundle, DVT: deep venous thrombosis.

**Table 3:** Effect of educational level on participants’ responses expressing their knowledge of and adherence to EBGs for VAP prevention.

Questions	Participants’ Responses	Highest Educational Level				*p* Value
		Diploma (*n* = 1)	Bachelor’s Degree (*n* = 69)	Master’s Degree (*n* = 9)	Doctorate (*n* = 7)	
**The first section; knowledge of EBGs for VAP prevention**						
Q1: Oral vs. nasal route for endotracheal intubation	Oral intubation is preferred over nasal route for VAP prevention	1 (100%)	48 (69.57%)	7 (77.78%)	2 (28.57%)	0.182
	Nasal intubation is preferred over oral route for VAP prevention	0 (0%)	7 (10.14%)	1 (11.11%)	1 (14.29%)	
	No difference in VAP incidence between both route	0 (0%)	8 (11.59%)	1 (11.11%)	4 (57.14%)	
	I don’t know	0 (0%)	6 (8.7%)	0 (0%)	0 (0%)	
Q2: Which of the following is not a standard component of the VB?	Head-end elevation	0 (0%)	4 (5.8%)	0 (0%)	0 (0%)	0.396
	Peptic ulcer prophylaxis	0 (0%)	1 (1.45%)	1 (11.11%)	0 (0%)	
	DVT prophylaxis	0 (0%)	3 (4.35%)	2 (22.22%)	2 (28.57%)	
	Nebulization with antibiotic	1 (100%)	58 (84.06%)	6 (66.67%)	5 (71.43%)	
	Daily sedation vacation and assessment of readiness to extubate	0 (0%)	3 (4.35%)	0 (0%)	0 (0%)	
Q3: Which of the following does not increase the incidence of VAP?	Intermittent sedation	1 (100%)	36 (52.17%)	5 (55.56%)	7 (100%)	0.051
	Aspiration of oropharyngeal and gastric secretion	0 (0%)	0 (0%)	1 (11.11%)	0 (0%)	
	Reduce clinical staffing	0 (0%)	21 (30.43%)	3 (33.33%)	0 (0%)	
	Prolonged mechanical ventilation	0 (0%)	12 (17.39%)	0 (0%)	0 (0%)	
Q4: To reduce the risk of VAP, the head end of the bed should be elevated to:	5–10°	0 (0%)	0 (0%)	1 (11.11%)	0 (0%)	<0.001 *
	10–20°	0 (0%)	0 (0%)	0 (0%)	2 (28.57%)	
	20–30°	0 (0%)	3 (4.35%)	2 (22.22%)	0 (0%)	
	30–45°	1 (100%)	66 (95.65%)	6 (66.67%)	5 (71.43%)	
Q5: How often should you attempt awakening and spontaneous breathing trials?	Every 6 h	0 (0%)	3 (4.35%)	0 (0%)	0 (0%)	0.342
	Every 12 h	0 (0%)	3 (4.35%)	0 (0%)	0 (0%)	
	Daily	1 (100%)	63 (91.3%)	8 (88.89%)	7 (100%)	
	Never	0 (0%)	0 (0%)	1 (11.11%)	0 (0%)	
Q6: How often should you perform hand hygiene?	before and after contact with the ventilator or ventilator circuit	0 (0%)	0 (0%)	1 (11.11%)	0 (0%)	0.155
	before and after touching the patient	0 (0%)	5 (7.25%)	1 (11.11%)	1 (14.29%)	
	all the above	1 (100%)	64 (92.75%)	7 (77.78%)	6 (85.71%)	
Q7: Endotracheal tubes with extra lumen for drainage of subglottic secretions	These endotracheal tubes reduce the risk of VAP	0 (0%)	49 (71.01%)	9 (100%)	4 (57.14%)	<0.001 *
	These endotracheal tubes increase the risk of VAP	0 (0%)	13 (18.84%)	0 (0%)	1 (14.29%)	
	These endotracheal tubes don’t influence the risk of VAP	1 (100%)	2 (2.9%)	0 (0%)	0 (0%)	
	I don’t know	0 (0%)	5 (7.25%)	0 (0%)	2 (28.57%)	
Q8: Kinetic vs. standard beds	Kinetic beds increase the risk of VAP	0 (0%)	6 (8.7%)	0 (0%)	0 (0%)	0.442
	Kinetic beds reduce the risk of VAP	0 (0%)	38 (55.07%)	7 (77.78%)	6 (85.71%)	
	The use of kinetic beds doesn’t influence the risk of VAP	0 (0%)	10 (14.49%)	1 (11.11%)	1 (14.29%)	
	I don’t know	1 (100%)	15 (21.74%)	1 (11.11%)	0 (0%)	
Q9: Patient positioning	Supine positioning is preferred for VAP prevention	0 (0%)	5 (7.25%)	0 (0%)	0 (0%)	0.935
	Semi-recumbent positioning is preferred for VAP prevention	1 (100%)	53 (76.81%)	8 (88.89%)	7 (100%)	
	The position of the pt doesn’t influence the risk of VAP	0 (0%)	7 (10.14%)	1 (11.11%)	0 (0%)	
	I don’t know	0 (0%)	4 (5.8%)	0 (0%)	0 (0%)	
Q10: Usage of 0.12% chlorhexidine gluconate antiseptic oral rinse	0.12% chlorhexidine oral rinse reduces the risk of VAP	0 (0%)	46 (66.67%)	9 (100%)	4 (57.14%)	0.019 *
	0.12% chlorhexidine oral rinse increases the risk of VAP	0 (0%)	7 (10.14%)	0 (0%)	0 (0%)	
	0.12% chlorhexidine oral rinse doesn’t influence the risk of VAP	0 (0%)	9 (13.04%)	0 (0%)	3 (42.86%)	
	I don’t know	1 (100%)	7 (10.14%)	0 (0%)	0 (0%)	
**The Second section; Adherence to the VB in daily practice**						
Q1: I always comply with the Institutional VB.	Yes	1 (100%)	63 (91.3%)	9 (100%)	7 (100%)	0.662
	No	0 (0%)	6 (8.7%)	0 (0%)	0 (0%)	
Q2: I adhere to existing oral care protocol.	Yes	1 (100%)	65 (94.2%)	9 (100%)	7 (100%)	0.793
	No	0 (0%)	4 (5.8%)	0 (0%)	0 (0%)	
Q3: I always use chlorhexidine oral rinse as recommended.	Yes	1 (100%)	56 (81.16%)	8 (88.89%)	5 (71.43%)	0.795
	No	0 (0%)	13 (18.84%)	1 (11.11%)	2 (28.57%)	
Q4: I assess the depth of sedation as often as recommended.	Yes	1 (100%)	67 (97.1%)	9 (100%)	7 (100%)	0.918
	No	0 (0%)	2 (2.9%)	0 (0%)	0 (0%)	
Q5: I interrupt continuous sedative infusions as recommended.	Yes	1 (100%)	65 (94.2%)	9 (100%)	7 (100%)	0.793
	No	0 (0%)	4 (5.8%)	0 (0%)	0 (0%)	
Q6: I perform spontaneous breathing test as recommended.	Yes	1 (100%)	65 (94.2%)	9 (100%)	7 (100%)	0.793
	No	0 (0%)	4 (5.8%)	0 (0%)	0 (0%)	
Q7: I always keep head of bed elevated to 30–45°.	Yes	1 (100%)	69 (100%)	9 (100%)	7 (100%)	----
	No	0 (0%)	0 (0%)	0 (0%)	0 (0%)	
Q8: I always make sure that mechanical DVT prophylaxis is inserted as recommended.	Yes	1 (100%)	69 (100%)	9 (100%)	7 (100%)	----
	No	0 (0%)	0 (0%)	0 (0%)	0 (0%)	
Q9: I always give pharmacological DVT prophylaxis as recommended.	Yes	1 (100%)	69 (100%)	9 (100%)	7 (100%)	-----
	No	0 (0%)	0 (0%)	0 (0%)	0 (0%)	
Q10: I always give pharmacological peptic ulcer prophylaxis as recommended.	Yes	1 (100%)	68 (98.55%)	9 (100%)	7 (100%)	0.969
	No	0 (0%)	1 (1.45%)	0 (0%)	0 (0%)	

*: statistically significant as *p* value < 0.05. VAP: ventilator-associated pneumonia, VB: ventilator bundle, DVT: deep venous thrombosis.

**Table 4:** Effect of years of experience on participants’ responses expressing their knowledge of and adherence to EBGs for VAP prevention.

Questions	Participants’ Responses	Years of Experience					*p* Value
		<1 Year (*n* = 9)	1–3 Years (*n* = 17)	4–6 Years (*n* = 25)	7–9 Years (*n* = 20)	>10 Years (*n* = 15)	
**The first section; knowledge of EBGs for VAP prevention**							
Q1: Oral vs. nasal route for endotracheal intubation	Oral intubation is preferred over nasal route for VAP prevention	4 (44.44%)	11 (64.71%)	21 (84%)	14 (70%)	8 (53.33%)	0.089
	Nasal intubation is preferred over oral route for VAP prevention	0 (0%)	1 (5.88%)	3 (12%)	2 (10%)	3 (20%)	
	No difference in VAP incidence between both route	3 (33.33%)	2 (11.76%)	1 (4%)	3 (15%)	4 (26.67%)	
	I don’t know	2 (22.22%)	3 (17.65%)	0 (0%)	1 (5%)	0 (0%)	
Q2: Which of the following is not a standard component of the VB?	Head-end elevation	2 (22.22%)	0 (0%)	2 (8%)	0 (0%)	0 (0%)	<0.001 *
	Peptic ulcer prophylaxis	0 (0%)	1 (5.88%)	0 (0%)	1 (5%)	0 (0%)	
	DVT prophylaxis	3 (33.33%)	0 (0%)	0 (0%)	1 (5%)	3 (20%)	
	Nebulization with antibiotic	2 (22.22%)	15 (88.24%)	23 (92%)	18 (90%)	12 (80%)	
	Daily sedation vacation and assessment of readiness to extubate	2 (22.22%)	1 (5.88%)	0 (0%)	0 (0%)	0 (0%)	
Q3: Which of the following does not increase the incidence of VAP?	Intermittent sedation	3 (33.33%)	11 (64.71%)	11 (44%)	13 (65%)	11 (73.33%)	0.053
	Aspiration of oropharyngeal and gastric secretion	0 (0%)	0 (0%)	1 (4%)	0 (0%)	0 (0%)	
	Reduce clinical staffing	1 (11.11%)	5 (29.41%)	9 (36%)	6 (30%)	3 (20%)	
	Prolonged mechanical ventilation	5 (55.56%)	1 (5.88%)	4 (16%)	1 (5%)	1 (6.67%)	
Q4: To reduce the risk of VAP, the head end of the bed should be elevated to:	5–10°	0 (0%)	0 (0%)	0 (0%)	1 (5%)	0 (0%)	0.209
	10–20°	0 (0%)	0 (0%)	0 (0%)	0 (0%)	2 (13.33%)	
	20–30°	0 (0%)	0 (0%)	2 (8%)	2 (10%)	1 (6.67%)	
	30–45°	9 (100%)	17 (100%)	23 (92%)	17 (85%)	12 (80%)	
Q5: How often should you attempt awakening and spontaneous breathing trials?	Every 6 h	3 (33.33%)	0 (0%)	0 (0%)	0 (0%)	0 (0%)	<0.001 *
	Every 12 h	0 (0%)	2 (11.76%)	1 (4%)	0 (0%)	0 (0%)	
	Daily	6 (66.67%)	15 (88.24%)	24 (96%)	19 (95%)	15 (100%)	
	Never	0 (0%)	0 (0%)	0 (0%)	1 (5%)	0 (0%)	
Q6: How often should you perform hand hygiene?	before and after contact with the ventilator or ventilator circuit	0 (0%)	0 (0%)	0 (0%)	0 (0%)	1 (6.67%)	0.539
	before and after touching the patient	0 (0%)	1 (5.88%)	2 (8%)	3 (15%)	1 (6.67%)	
	all the above	9 (100%)	16 (94.12%)	23 (92%)	17 (85%)	13 (86.67%)	
Q7: Endotracheal tubes with extra lumen for drainage of subglottic secretions	These endotracheal tubes reduce the risk of VAP	4 (44.44%)	12 (70.59%)	21 (84%)	14 (70%)	11 (73.33%)	0.038 *
	These endotracheal tubes increase the risk of VAP	5 (55.56%)	2 (11.76%)	2 (8%)	4 (20%)	1 (6.67%)	
	These endotracheal tubes don’t influence the risk of VAP	0 (0%)	1 (5.88%)	0 (0%)	2 (10%)	0 (0%)	
	I don’t know	0 (0%)	2 (11.76%)	2 (8%)	0 (0%)	3 (20%)	
Q8: Kinetic vs. standard beds	Kinetic beds increase the risk of VAP	0 (0%)	2 (11.76%)	3 (12%)	1 (5%)	0 (0%)	0.005 *
	Kinetic beds reduce the risk of VAP	3 (33.33%)	9 (52.94%)	15 (60%)	14 (70%)	10 (66.67%)	
	The use of kinetic beds doesn’t influence the risk of VAP	0 (0%)	1 (5.88%)	2 (8%)	4 (20%)	5 (33.33%)	
	I don’t know	6 (66.67%)	5 (29.41%)	5 (20%)	1 (5%)	0 (0%)	
Q9: Patient positioning	Supine positioning is preferred for VAP prevention	0 (0%)	2 (11.76%)	2 (8%)	0 (0%)	1 (6.67%)	0.062
	Semi-recumbent positioning is preferred for VAP prevention	5 (55.56%)	12 (70.59%)	19 (76%)	19 (95%)	14 (93.33%)	
	The position of the pt doesn’t influence the risk of VAP	3 (33.33%)	3 (17.65%)	1 (4%)	1 (5%)	0 (0%)	
	I don’t know	1 (11.11%)	0 (0%)	3 (12%)	0 (0%)	0 (0%)	
Q10: Usage of 0.12% chlorhexidine gluconate antiseptic oral rinse	0.12% chlorhexidine oral rinse reduces the risk of VAP	3 (33.33%)	14 (82.35%)	18 (72%)	12 (60%)	12 (80%)	0.002 *
	0.12% chlorhexidine oral rinse increases the risk of VAP	0 (0%)	1 (5.88%)	3 (12%)	3 (15%)	0 (0%)	
	0.12% chlorhexidine oral rinse doesn’t influence the risk of VAP	1 (11.11%)	2 (11.76%)	3 (12%)	3 (15%)	3 (20%)	
	I don’t know	5 (55.56%)	0 (0%)	1 (4%)	2 (10%)	0 (0%)	
**The Second section; Adherence to the VB in daily practice**							
Q1: I always comply with the Institutional VB.	Yes	6 (66.67%)	17 (100%)	24 (96%)	18 (90%)	15 (100%)	0.013 *
	No	3 (33.33%)	0 (0%)	1 (4%)	2 (10%)	0 (0%)	
Q2: I adhere to existing oral care protocol.	Yes	7 (77.78%)	17 (100%)	25 (100%)	18 (90%)	15 (100%)	0.035 *
	No	2 (22.22%)	0 (0%)	0 (0%)	2 (10%)	0 (0%)	
Q3: I always use chlorhexidine oral rinse as recommended.	Yes	5 (55.56%)	14 (82.35%)	21 (84%)	18 (90%)	12 (80%)	0.278
	No	4 (44.44%)	3 (17.65%)	4 (16%)	2 (10%)	3 (20%)	
Q4: I assess the depth of sedation as often as recommended.	Yes	7 (77.78%)	17 (100%)	25 (100%)	20 (100%)	15 (100%)	0.002 *
	No	2 (22.22%)	0 (0%)	0 (0%)	0 (0%)	0 (0%)	
Q5: I interrupt continuous sedative infusions as recommended.	Yes	5 (55.56%)	17 (100%)	25 (100%)	20 (100%)	15 (100%)	<0.001 *
	No	4 (44.44%)	0 (0%)	0 (0%)	0 (0%)	0 (0%)	
Q6: I perform spontaneous breathing test as recommended.	Yes	5 (55.56%)	17 (100%)	25 (100%)	20 (100%)	15 (100%)	<0.001 *
	No	4 (44.44%)	0 (0%)	0 (0%)	0 (0%)	0 (0%)	
Q7: I always keep head of bed elevated to 30–45°.	Yes	9 (100%)	17 (100%)	25 (100%)	20 (100%)	15 (100%)	----
	No	0 (0%)	0 (0%)	0 (0%)	0 (0%)	0 (0%)	
Q8: I always make sure that mechanical DVT prophylaxis is inserted as recommended.	Yes	9 (100%)	17 (100%)	25 (100%)	20 (100%)	15 (100%)	----
	No	0 (0%)	0 (0%)	0 (0%)	0 (0%)	0 (0%)	
Q9: I always give pharmacological DVT prophylaxis as recommended.	Yes	9 (100%)	17 (100%)	25 (100%)	20 (100%)	15 (100%)	-----
	No	0 (0%)	0 (0%)	0 (0%)	0 (0%)	0 (0%)	
Q10: I always give pharmacological peptic ulcer prophylaxis as recommended.	Yes	8 (88.89%)	17 (100%)	25 (100%)	20 (100%)	15 (100%)	0.070
	No	1 (11.11%)	0 (0%)	0 (0%)	0 (0%)	0 (0%)	

*: statistically significant as *p* value < 0.05. VAP: ventilator-associated pneumonia, VB: ventilator bundle, DVT: deep venous thrombosis.

**Table 5:** Effect of job titles on participants’ responses expressing their knowledge of and adherence to EBGs for VAP prevention.

Questions	Participants’ Responses	Job Titles			*p* Value
		Physician (*n* = 18)	Nurse (*n* = 57)	Respiratory Therapist (*n* = 11)	
**The first section; knowledge of EBGs for VAP prevention**					
Q1: Oral vs. nasal route for endotracheal intubation	Oral intubation is preferred over nasal route for VAP prevention	12 (66.67%)	41 (71.93%)	5 (45.45%)	0.116
	Nasal intubation is preferred over oral route for VAP prevention	2 (11.11%)	5 (8.77%)	2 (18.18%)	
	No difference in VAP incidence between both route	4 (22.22%)	5 (8.77%)	4 (36.36%)	
	I don’t know	0 (0%)	6 (10.53%)	0 (0%)	
Q2: Which of the following is not a standard component of the VB?	Head-end elevation	4 (22.22%)	0 (0%)	0 (0%)	<0.001 *
	Peptic ulcer prophylaxis	1 (5.56%)	1 (1.75%)	0 (0%)	
	DVT prophylaxis	4 (22.22%)	3 (5.26%)	0 (0%)	
	Nebulization with antibiotic	9 (50%)	50 (87.72%)	11 (100%)	
	Daily sedation vacation and assessment of readiness to extubate	0 (0%)	3 (5.26%)	0 (0%)	
Q3: Which of the following does not increase the incidence of VAP?	Intermittent sedation	12 (66.67%)	31 (54.39%)	6 (54.55%)	0.119
	Aspiration of oropharyngeal and gastric secretion	0 (0%)	0 (0%)	1 (9.09%)	
	Reduce clinical staffing	3 (16.67%)	17 (29.82%)	4 (36.36%)	
	Prolonged mechanical ventilation	3 (16.67%)	9 (15.79%)	0 (0%)	
Q4: To reduce the risk of VAP, the head end of the bed should be elevated to:	5–10°	1 (5.56%)	0 (0%)	0 (0%)	0.007 *
	10–20°	2 (11.11%)	0 (0%)	0 (0%)	
	20–30°	2 (11.11%)	1 (1.75%)	2 (18.18%)	
	30–45°	13 (72.22%)	56 (98.25%)	9 (81.82%)	
Q5: How often should you attempt awakening and spontaneous breathing trials?	Every 6 h	2 (11.11%)	1 (1.75%)	0 (0%)	0.149
	Every 12 h	0 (0%)	2 (3.51%)	1 (9.09%)	
	Daily	15 (83.33%)	54 (94.74%)	10 (90.91%)	
	Never	1 (5.56%)	0 (0%)	0 (0%)	
Q6: How often should you perform hand hygiene?	Before and after contact with the ventilator or ventilator circuit	1 (5.56%)	0 (0%)	0 (0%)	0.032 *
	Before and after touching the patient	4 (22.22%)	3 (5.26%)	0 (0%)	
	all the above	13 (72.22%)	54 (94.74%)	11 (100%)	
Q7: Endotracheal tubes with extra lumen for drainage of subglottic secretions	These endotracheal tubes reduce the risk of VAP	14 (77.78%)	38 (66.67%)	10 (90.91%)	0.544
	These endotracheal tubes increase the risk of VAP	1 (5.56%)	12 (21.05%)	1 (9.09%)	
	These endotracheal tubes don’t influence the risk of VAP	1 (5.56%)	2 (3.51%)	0 (0%)	
	I don’t know	2 (11.11%)	5 (8.77%)	0 (0%)	
Q8: Kinetic vs. standard beds	Kinetic beds increase the risk of VAP	0 (0%)	4 (7.02%)	2 (18.18%)	0.084
	Kinetic beds reduce the risk of VAP	15 (83.33%)	31 (54.39%)	5 (45.45%)	
	The use of kinetic beds doesn’t influence the risk of VAP	2 (11.11%)	10 (17.54%)	0 (0%)	
	I don’t know	1 (5.56%)	12 (21.05%)	4 (36.36%)	
Q9: Patient positioning	Supine positioning is preferred for VAP prevention	0 (0%)	3 (5.26%)	2 (18.18%)	0.023 *
	Semi-recumbent positioning is preferred for VAP prevention	18 (100%)	44 (77.19%)	7 (63.64%)	
	The position of the pt doesn’t influence the risk of VAP	0 (0%)	8 (14.04%)	0 (0%)	
	I don’t know	0 (0%)	2 (3.51%)	2 (18.18%)	
Q10: Usage of 0.12% chlorhexidine gluconate antiseptic oral rinse	0.12% chlorhexidine oral rinse reduces the risk of VAP	12 (66.67%)	38 (66.67%)	9 (81.82%)	0.912
	0.12% chlorhexidine oral rinse increases the risk of VAP	2 (11.11%)	5 (8.77%)	0 (0%)	
	0.12% chlorhexidine oral rinse doesn’t influence the risk of VAP	3 (16.67%)	8 (14.04%)	1 (9.09%)	
	I don’t know	1 (5.56%)	6 (10.53%)	1 (9.09%)	
**The Second section; Adherence to the VB in daily practice**					
Q1: I always comply with the Institutional VB.	Yes	18 (100%)	53 (92.98%)	9 (81.82%)	0.176
	No	0 (0%)	4 (7.02%)	2 (18.18%)	
Q2: I adhere to existing oral care protocol.	Yes	18 (100%)	53 (92.98%)	11 (100%)	0.344
	No	0 (0%)	4 (7.02%)	0 (0%)	
Q3: I always use chlorhexidine oral rinse as recommended.	Yes	14 (77.78%)	47 (82.46%)	9 (81.82%)	0.905
	No	4 (22.22%)	10 (17.54%)	2 (18.18%)	
Q4: I assess the depth of sedation as often as recommended.	Yes	18 (100%)	55 (96.49%)	11 (100%)	0.594
	No	0 (0%)	2 (3.51%)	0 (0%)	
Q5: I interrupt continuous sedative infusions as recommended.	Yes	18 (100%)	54 (94.74%)	10 (90.91%)	0.493
	No	0 (0%)	3 (5.26%)	1 (9.09%)	
Q6: I perform spontaneous breathing test as recommended.	Yes	18 (100%)	54 (94.74%)	10 (90.91%)	0.493
	No	0 (0%)	3 (5.26%)	1 (9.09%)	
Q7: I always keep head of bed elevated to 30–45°.	Yes	18 (100%)	57 (100%)	11 (100%)	----
	No	0 (0%)	0 (0%)	0 (0%)	
Q8: I always make sure that mechanical DVT prophylaxis is inserted as recommended.	Yes	18 (100%)	57 (100%)	11 (100%)	----
	No	0 (0%)	0 (0%)	0 (0%)	
Q9: I always give pharmacological DVT prophylaxis as recommended.	Yes	18 (100%)	57 (100%)	11 (100%)	----
	No	0 (0%)	0 (0%)	0 (0%)	
Q10: I always give pharmacological peptic ulcer prophylaxis as recommended.	Yes	18 (100%)	56 (98.25%)	11 (100%)	0.773
	No	0 (0%)	1 (1.75%)	0 (0%)	

*: statistically significant as *p* value < 0.05. VAP: ventilator-associated pneumonia, VB: ventilator bundle, DVT: deep venous thrombosis.

**Table 6:** Assessment of the correct knowledge of the studied participants regarding evidence-based VAP prevention guidelines according to their highest educational level.

Questions	Correct Knowledge	Total (*n* = 86)	Highest Educational Level				*p* Value
			Diploma (*n* = 1)	Bachelor’s Degree (*n* = 69)	Master’s Degree (*n* = 9)	Doctorate (*n* = 7)	
Q1: Oral vs. nasal route for endotracheal intubation	Yes	58 (67.4%)	1 (100%)	48 (69.6%)	7 (77.8%)	2 (28.6%)	0.118
	No	28 (32.6%)	0 (0%)	21 (30.4%)	2 (22.2%)	5 (71.4%)	
Q2: Which of the following is not a standard component of the VB	Yes	70 (81.4%)	1 (100%)	58 (84.1%)	6 (66.7%)	5 (71.4%)	0.513
	No	16 (18.6%)	0 (0%)	11 (15.9%)	3 (33.3%)	2 (28.6%)	
Q3: Which of the following does not increase the incidence of VAP	Yes	49 (57%)	1 (100%)	36 (52.2%)	5 (55.6%)	7 (100%)	0.082
	No	37 (43%)	0 (0%)	33 (47.8%)	4 (44.4%)	0 (0%)	
Q4: Degree of elevation to the head end of the bed to reduce the risk of VAP	Yes	78 (90.7%)	1 (100%)	66 (95.7%)	6 (66.7%)	5 (71.4%)	0.010 *
	No	8 (9.3%)	0 (0%)	3 (4.3%)	3 (33.3%)	2 (28.6%)	
Q5: Attempt awakening and spontaneous breathing trials	Yes	79 (91.9%)	1 (100%)	63 (91.3%)	8 (88.9%)	7 (100%)	0.839
	No	7 (8.1%)	0 (0%)	6 (8.7%)	1 (11.1%)	0 (0%)	
Q6: Perform hand hygiene	Yes	78 (90.7%)	1 (100%)	64 (92.8%)	7 (77.8%)	6 (85.7%)	0.487
	No	8 (9.3%)	0 (0%)	5 (7.2%)	2 (22.2%)	1 (14.3%)	
Q7: Endotracheal tubes with extra lumen for drainage of subglottic secretions	Yes	62 (72.1%)	0 (0%)	49 (71%)	9 (100%)	4 (57.1%)	0.076
	No	24 (27.9%)	1 (100%)	20 (29%)	0 (0%)	3 (42.9%)	
Q8: Kinetic vs. standard beds	Yes	12 (14%)	0 (0%)	10 (14.5%)	1 (11.1%)	1 (14.3%)	0.971
	No	74 (86%)	1 (100%)	59 (85.5%)	8 (88.9%)	6 (85.7%)	
Q9: Patient positioning	Yes	69 (80.2%)	1 (100%)	53 (76.8%)	8 (88.9%)	7 (100%)	0.406
	No	17 (19.8%)	0 (0%)	16 (23.2%)	1 (11.1%)	0 (0%)	
Q10: Use of 0.12% chlorhexidine gluconate antiseptic oral rinse	Yes	59 (68.6%)	0 (0%)	46 (66.7%)	9 (100%)	4 (57.1%)	0.077
	No	27 (31.4%)	1 (100%)	23 (33.3%)	0 (0%)	3 (42.9%)	

*: statistically significant as *p* value < 0.05. VAP: ventilator-associated pneumonia, VB: ventilator bundle.

**Table 7:** Assessment of the correct knowledge of the studied participants regarding evidence-based VAP prevention guidelines according to their years of experience.

Questions	Correct Knowledge	Total (*n* = 86)	Years of Experience					*p* Value
			<1 Year (*n* = 9)	1–3 Years (*n* = 17)	4–6 Years (*n* = 25)	7–9 Years (*n* = 20)	>10 Years (*n* = 15)	
Q1: Oral vs. nasal route for endotracheal intubation	Yes	58 (67.4%)	4 (44.4%)	11 (64.7%)	21 (84%)	14 (70%)	8 (53.3%)	0.149
	No	28 (32.6%)	5 (55.6%)	6 (35.3%)	4 (16%)	6 (30%)	7 (46.7%)	
Q2: Which of the following is not a standard component of the VB	Yes	70 (81.4%)	2 (22.2%)	15 (88.2%)	23 (92%)	18 (90%)	12 (80%)	<0.001 *
	No	16 (18.6%)	7 (77.8%)	2 (11.8%)	2 (8%)	2 (10%)	3 (20%)	
Q3: Which of the following does not increase the incidence of VAP	Yes	49 (57%)	3 (33.3%)	11 (64.7%)	11 (44%)	13 (65%)	11 (73.3%)	0.175
	No	37 (43%)	6 (66.7%)	6 (35.3%)	14 (56%)	7 (35%)	4 (26.7%)	
Q4: Degree of elevation to the head end of the bed to reduce the risk of VAP	Yes	78 (90.7%)	9 (100%)	17 (100%)	23 (92%)	17 (85%)	12 (80%)	0.238
	No	8 (9.3%)	0 (0%)	0 (0%)	2 (8%)	3 (15%)	3 (20%)	
Q5: Attempt awakening and spontaneous breathing trials	Yes	79 (91.9%)	6 (66.7%)	15 (88.2%)	24 (96%)	19 (95%)	15 (100%)	0.039 *
	No	7 (8.1%)	3 (33.3%)	2 (11.8%)	1 (4%)	1 (5%)	0 (0%)	
Q6: Perform hand hygiene	Yes	78 (90.7%)	9 (100%)	16 (94.1%)	23 (92%)	17 (85%)	13 (86.7%)	0.687
	No	8 (9.3%)	0 (0%)	1 (5.9%)	2 (8%)	3 (15%)	2 (13.3%)	
Q7: Endotracheal tubes with extra lumen for drainage of subglottic secretions	Yes	62 (72.1%)	4 (44.4%)	12 (70.6%)	21 (84%)	14 (70%)	11 (73.3%)	0.262
	No	24 (27.9%)	5 (55.6%)	5 (29.4%)	4 (16%)	6 (30%)	4 (26.7%)	
Q8: Kinetic vs. standard beds	Yes	12 (14%)	0 (0%)	1 (5.9%)	2 (8%)	4 (20%)	5 (33.3%)	0.077
	No	74 (86%)	9 (100%)	16 (94.1%)	23 (92%)	16 (80%)	10 (66.7%)	
Q9: Patient positioning	Yes	69 (80.2%)	5 (55.6%)	12 (70.6%)	19 (76%)	19 (95%)	14 (93.3%)	0.058
	No	17 (19.8%)	4 (44.4%)	5 (29.4%)	6 (24%)	1 (5%)	1 (6.7%)	
Q10: Use of 0.12% chlorhexidine gluconate antiseptic oral rinse	Yes	59 (68.6%)	3 (33.3%)	14 (82.4%)	18 (72%)	12 (60%)	12 (80%)	0.077
	No	27 (31.4%)	6 (66.7%)	3 (17.6%)	7 (28%)	8 (40%)	3 (20%)	

*: statistically significant as *p* value < 0.05. VAP: ventilator-associated pneumonia, VB: ventilator bundle.

**Table 8:** Assessment of the correct knowledge of the studied participants regarding evidence-based VAP prevention guidelines according to their job title.

Questions	Correct Knowledge	Total (*n* = 86)	Job Titles			*p* Value
			Physician (*n* = 18)	Nurse (*n* = 57)	Respiratory Therapist (*n* = 11)	
Q1: Oral vs. nasal route for endotracheal intubation	Yes	58 (67.4%)	12 (66.7%)	41 (71.9%)	5 (45.5%)	0.229
	No	28 (32.6%)	6 (33.3%)	16 (28.1%)	6 (54.5%)	
Q2: Which of the following is not a standard component of the VB	Yes	70 (81.4%)	9 (50%)	50 (87.7%)	11 (100%)	<0.001 *
	No	16 (18.6%)	9 (50%)	7 (12.3%)	0 (0%)	
Q3: Which of the following does not increase the incidence of VAP	Yes	49 (57%)	12 (66.7%)	31 (54.4%)	6 (54.5%)	0.674
	No	37 (43%)	6 (33.3%)	26 (45.6%)	5 (45.5%)	
Q4: Degree of elevation to the head end of the bed to reduce the risk of VAP	Yes	78 (90.7%)	13 (72.2%)	56 (98.2%)	9 (81.8%)	0.002 *
	No	8 (9.3%)	5 (27.8%)	1 (1.8%)	2 (18.2%)	
Q5: Attempt awakening and spontaneous breathing trials	Yes	79 (91.9%)	15 (83.3%)	54 (94.7%)	10 (90.9%)	0.302
	No	7 (8.1%)	3 (16.7%)	3 (5.3%)	1 (9.1%)	
Q6: Perform hand hygiene	Yes	78 (90.7%)	13 (72.2%)	54 (94.7%)	11 (100%)	0.009 *
	No	8 (9.3%)	5 (27.8%)	3 (5.3%)	0 (0%)	
Q7: Endotracheal tubes with extra lumen for drainage of subglottic secretions	Yes	62 (72.1%)	14 (77.8%)	38 (66.7%)	10 (90.9%)	0.217
	No	24 (27.9%)	4 (22.2%)	19 (33.3%)	1 (9.1%)	
Q8: Kinetic vs. standard beds	Yes	12 (14%)	2 (11.1%)	10 (17.5%)	0 (0%)	0.284
	No	74 (86%)	16 (88.9%)	47 (82.5%)	11 (100%)	
Q9: Patient positioning	Yes	69 (80.2%)	18 (100%)	44 (77.2%)	7 (63.6%)	0.035 *
	No	17 (19.8%)	0 (0%)	13 (22.8%)	4 (36.4%)	
Q10: Use of 0.12% chlorhexidine gluconate antiseptic oral rinse	Yes	59 (68.6%)	12 (66.7%)	38 (66.7%)	9 (81.8%)	0.600
	No	27 (31.4%)	6 (33.3%)	19 (33.3%)	2 (18.2%)	

*: statistically significant as *p* value < 0.05. VAP: ventilator-associated pneumonia, VB: ventilator bundle.

**Table 9:** Participants’ perceived barriers to implementation of the institutional VAP-prevention bundle.

Questions	Number of Participants	Barrier	Participants’ Responses	
			Yes	No
Q1: What barrier prevents you from always complying with the Institutional VB?	6	Lack of guidelines	0 (0%)	6 (100%)
		Lack of education	6 (100%)	0 (0%)
		Inadequate resources	0 (0%)	6 (100%)
		Disagreement with reported results	0 (0%)	6 (100%)
		Fear of potential adverse effects	0 (0%)	6 (100%)
		Patient discomfort	0 (0%)	6 (100%)
		Costs	0 (0%)	6 (100%)
Q2: What barrier prevents you from adhering to existing oral care protocol?	4	Lack of guidelines	0 (0%)	4 (100%)
		Lack of education	3 (75%)	1 (25%)
		Inadequate resources	0 (0%)	4 (100%)
		Disagreement with reported results	0 (0%)	4 (100%)
		Fear of potential adverse effects	0 (0%)	4 (100%)
		Patient discomfort	1 (25%)	3 (75%)
		Costs	0 (0%)	4 (100%)
Q3: What barrier prevents you from always using chlorhexidine oral rinse as recommended?	16	Lack of guidelines	0 (0%)	16 (100%)
		Lack of education	12 (75%)	4 (25%)
		Inadequate resources	0 (0%)	16 (100%)
		Disagreement with reported results	0 (0%)	16 (100%)
		Fear of potential adverse effects	4 (25%)	12 (75%)
		Patient discomfort	0 (0%)	16 (100%)
		Costs	0 (0%)	16 (100%)
Q4: What barrier prevents you from assessing the depth of sedation as often as recommended?	2	Lack of guidelines	2 (100%)	0 (0%)
		Lack of education	0 (0%)	2 (100%)
		Inadequate resources	0 (0%)	2 (100%)
		Disagreement with reported results	0 (0%)	2 (100%)
		Fear of potential adverse effects	0 (0%)	2 (100%)
		Patient discomfort	0 (0%)	2 (100%)
		Costs	0 (0%)	2 (100%)
Q5: What barrier prevents you from interrupting continuous sedative infusions as recommended?	4	Lack of guidelines	0 (0%)	4 (100%)
		Lack of education	0 (0%)	4 (100%)
		Inadequate resources	0 (0%)	4 (100%)
		Disagreement with reported results	0 (0%)	4 (100%)
		Fear of potential adverse effects	0 (0%)	4 (100%)
		Patient discomfort	4 (100%)	0 (0%)
		Costs	0 (0%)	4 (100%)
Q6: What barrier prevents you from performing spontaneous breathing test as recommended?	4	Lack of guidelines	0 (0%)	4 (100%)
		Lack of education	4 (100%)	0 (0%)
		Inadequate resources	0 (0%)	4 (100%)
		Disagreement with reported results	0 (0%)	4 (100%)
		Fear of potential adverse effects	0 (0%)	4 (100%)
		Patient discomfort	0 (0%)	4 (100%)
		Costs	0 (0%)	4 (100%)
Q7: What barrier prevents you from always keeping head of bed elevated to 30–45°?	0	Lack of guidelines	0 (0%)	0 (0%)
		Lack of education	0 (0%)	0 (0%)
		Inadequate resources	0 (0%)	0 (0%)
		Disagreement with reported results	0 (0%)	0 (0%)
		Fear of potential adverse effects	0 (0%)	0 (0%)
		Patient discomfort	0 (0%)	0 (0%)
		Costs	0 (0%)	0 (0%)
Q8: What barrier prevents you from always making sure that mechanical DVT prophylaxis is inserted as recommended?	0	Lack of guidelines	0 (0%)	0 (0%)
		Lack of education	0 (0%)	0 (0%)
		Inadequate resources	0 (0%)	0 (0%)
		Disagreement with reported results	0 (0%)	0 (0%)
		Fear of potential adverse effects	0 (0%)	0 (0%)
		Patient discomfort	0 (0%)	0 (0%)
		Costs	0 (0%)	0 (0%)
Q9: What barrier prevents you from always giving pharmacological DVT prophylaxis as recommended?	0	Lack of guidelines	0 (0%)	0 (0%)
		Lack of education	0 (0%)	0 (0%)
		Inadequate resources	0 (0%)	0 (0%)
		Disagreement with reported results	0 (0%)	0 (0%)
		Fear of potential adverse effects	0 (0%)	0 (0%)
		Patient discomfort	0 (0%)	0 (0%)
		Costs	0 (0%)	0 (0%)
Q10: What barrier prevents you from always giving pharmacological peptic ulcer prophylaxis as recommended?	1	Lack of guidelines	0 (0%)	1 (100%)
		Lack of education	0 (0%)	1 (100%)
		Inadequate resources	0 (0%)	1 (100%)
		Disagreement with reported results	0 (0%)	1 (100%)
		Fear of potential adverse effects	1 (100%)	0 (0%)
		Patient discomfort	0 (0%)	1 (100%)
		Costs	0 (0%)	1 (100%)

VB: ventilator bundle, DVT: deep venous thrombosis.

## Data Availability

All data are available in the manuscript.

## Abbreviations

The following abbreviations are used in this manuscript:

ARDS: adult respiratory distress syndrome; ASHP: American society of health-system pharmacists; CDC: Centers for Disease Control; CFUs: colony-forming units; CT: computed tomography; DVT: deep vein thrombosis; EBGs: evidence-based guidelines; FiO_2_: fraction of inspired oxygen; HAI: healthcare-associated infection; HOB: head-of-bed; ICU: intensive care unit; IDSA: Infectious Diseases Society of America; IRB: Institutional Research Board; IVAC: infection-related ventilator-associated condition; PEEP: positive end-expiratory pressure; SAT: spontaneous awakening trial; SBT: spontaneous breathing trial; SSD: subglottic secretion drainage; SUP: stress ulcer prophylaxis; VAC: ventilator-associated condition; VAEs: ventilator-associated events; VAP: ventilator-associated pneumonia; VB: ventilator bundle; VTE: venous thromboembolism; WBCs: white blood cells.
